# Reconstruction after Talar Tumor Resection: A Systematic Review

**DOI:** 10.3390/curroncol29120769

**Published:** 2022-12-12

**Authors:** Shinji Tsukamoto, Andreas F. Mavrogenis, Kanya Honoki, Akira Kido, Yuu Tanaka, Hiromasa Fujii, Yoshinori Takakura, Yasuhito Tanaka, Costantino Errani

**Affiliations:** 1 Department of Orthopaedic Surgery, Nara Medical University, Nara 634-8521, Japan; 2 First Department of Orthopaedics, National and Kapodistrian University of Athens, School of Medicine, 11527 Athens, Greece; 3 Department of Rehabilitation Medicine, Nara Medical University, Nara 634-8521, Japan; 4 Department of Rehabilitation Medicine, Wakayama Professional University of Rehabilitation, Wakayama 640-8222, Japan; 5 Department of Orthopaedic Surgery, Nishi Nara Central Hospital, Nara 631-0022, Japan; 6 Orthopaedic Oncology Service, IRCCS Istituto Ortopedico Rizzoli, 40136 Bologna, Italy

**Keywords:** talus, tumor, reconstruction, talar prosthesis, tibiocalcaneal fusion, arthrodesis

## Abstract

This systematic review investigated the functional outcomes and complications of reconstruction methods after talar tumor resection. A systematic search of PubMed, Embase, and the Cochrane Central Register of Controlled Trials databases identified 156 studies, of which 20 (23 patients) were ultimately included. The mean Musculoskeletal Tumor Society scores in the groups reconstructed using tibiocalcaneal fusion (*n* = 17), frozen autograft (*n* = 1), and talar prosthesis (*n* = 5) were 77.6 (range 66–90), 70, and 90 (range 87–93), respectively. Regarding complications, sensory deficits were observed in one patient (6%) and venous thrombosis in two patients (12%) in the tibiocalcaneal fusion group, while osteoarthritis was observed in one patient (100%) in the frozen autograft group. No complications were observed in the talar prosthesis group. Reconstruction with talar prosthesis seems preferable to conventional tibiocalcaneal fusion after talar tumor resection because it offers better function and fewer complications. However, as this systematic review included only retrospective studies with a small number of patients, its results require re-evaluation in future randomized controlled trials with larger numbers of patients.

## 1. Introduction

Bone tumors arising in the foot account for 1–2% of all bone tumors [1]. Approximately 8–15% of foot tumors occur in the talus [2,3,4]. Young et al. reported that among 23 talar tumors, 17 (74%) were benign, 3 (13%) were intermediate, and 3 (13%) were malignant [5]. The breakdown is osteoid osteoma (7 cases), intraosseous ganglion (2 cases), osteochondroma (2 cases), membranous lipodystrophy (1 case), chondroblastoma (4 cases), desmoplastic fibroma (1 case), osteoblastoma (2 cases), giant cell tumor of bone (1 case), chondrosarcoma (2 cases), and osteosarcoma (1 case) [5]. The patients with benign talar tumors presented at a mean age of 25 years (range 10–47 years) and those with malignant talar tumors at 49 years (range 39–64 years) [5]. Murari et al. reported 17 primary talar tumors, including osteoblastoma (10 cases), chondromyxoid fibroma (2 cases), chondroblastoma (2 cases), giant cell tumor of bone (1 case), bone cyst (1 case), osteochondroma (1 case), and osteoid osteoma (1 case) [3]. Dhillon et al. reported 12 primary talar tumors, including giant cell tumor of bone (5 cases), osteochondroma (2 cases), osteoblastoma (2 cases), Ewing’s sarcoma (2 cases), and osteoid osteoma (1 case) [6].

The previous standard surgical procedure for talar malignant tumors was below-the-knee amputation. However, with the development of effective chemotherapy agents, treatment plans, and modern surgical techniques, wide resection and limb salvage have become feasible for talar malignant tumors [7,8,9,10,11,12,13]. Patients with sarcoma of the lower extremities who undergo limb salvage reportedly have higher Musculoskeletal Tumor Society (MSTS) scores than those who undergo amputation [14,15,16]. In patients with intermediate-grade tumors, such as osteoblastoma, giant cell tumor of the bone, epithelioid hemangioma or desmoplastic fibroma with large extraskeletal lesions, poor residual cortical bone, and curettage difficulty, marginal tumor resection is required [6,17,18,19,20,21,22,23,24,25,26]. A variety of reconstruction techniques have been described for treating bone defects after talar tumor resection, including tibiocalcaneal fusion [7,9,10,13,17,18,19,20,21,22,25,27,28,29], reconstruction with a frozen autograft [8], and talar prostheses [11,12,23,24,30]. However, owing to their rarity, the results of each reconstruction method are not well known as they are mostly case reports. Naturally, there have been no randomized controlled trials (RCTs). Therefore, the optimal reconstruction method remains unknown. To the best of our knowledge, no systematic review of the literature has illustrated this issue.

To investigate the functional outcomes and complications of reconstruction methods after the resection of tumors originating in the talus, we performed this systematic review of studies that reported the postoperative function and complications of reconstruction after resection.

## 2. Materials and Methods

This study followed the recommendations of the Preferred Reporting Items for Systematic Reviews and Meta-analyses 2020 statement [31]. The study protocol was registered in the UMIN Clinical Trials Registration as UMIN000047105 (http://www.umin.ac.jp/ctr/index.htm (accessed on 7 March 2022)). The study was conducted in accordance with the guidelines of the Declaration of Helsinki and approved by the Institutional Review Board (or Ethics Committee) of Nara Medical University (protocol code 2833 and date of approval 27 November 2020). The requirement for written consent from participants at Nara Medical University was waived because an “opt-out” process was used and the retrospective nature of the study.

### 2.1. Eligibility Criteria

Only studies reporting on the postoperative function and complications after talar tumor resection followed by reconstruction were included. Patients who underwent resection alone for talar tumors without reconstruction or amputation were excluded. We also excluded cases for which the postoperative function and complications were not specified. Patients with talar tumors treated with curettage or partial resection were excluded. The data were extracted for the ankle joint range of motion (ROM) and American Orthopedic Foot and Ankle Society (AOFAS) ankle-hindfoot score, in which 40 points represents pain and 60 points represents ankle-hindfoot function and alignment, MSTS score [32], leg length difference, and complications. Only English and Japanese studies were included, and no restrictions were placed on the year of publication.

### 2.2. Literature Search and Study Selection

The literature was searched on 19 January 2022, using a systematic search strategy in PubMed, Embase, and the Cochrane Central Register of Controlled Trials (Table S1). The reference lists of the retrieved studies were manually searched to identify other relevant studies.

### 2.3. Data Collection and Presentation

Two authors (S.T. and A.K.) independently selected the studies and extracted the data. In cases of disagreement, agreement was reached or a third author was consulted. Data were collected using a data collection sheet and included: author, year of publication, journal name, study type, patient age, patient sex, tumor histological type, reconstruction method, ankle joint ROM, postoperative function of the affected limb (AOFAS and MSTS scores), leg length difference, complications, oncological outcome, and postoperative follow-up period.

### 2.4. Data Summary

Table 1 and Table 2 summarize the data extracted from the included studies.

Table 3 summarizes the age, sex, tumor histology, ankle ROM, AOFAS and MSTS scores, leg length difference, complications, oncologic outcomes, and follow-up period for each reconstruction method (tibiocalcaneal fusion, frozen autograft reconstruction, and talar prosthesis reconstruction).

### 2.5. Assessment of Methodological Quality

Two authors (ST and AK) independently assessed the quality of the included studies. In cases of disagreement, agreement was reached or a third author was consulted. The articles included in the final analysis were independently assessed according to the Risk of Bias Assessment tool for Non-randomized Studies (RoBANS) tool to assess the quality of non-randomized studies in meta-studies [33].

### 2.6. Search Results

**Figure 1:**
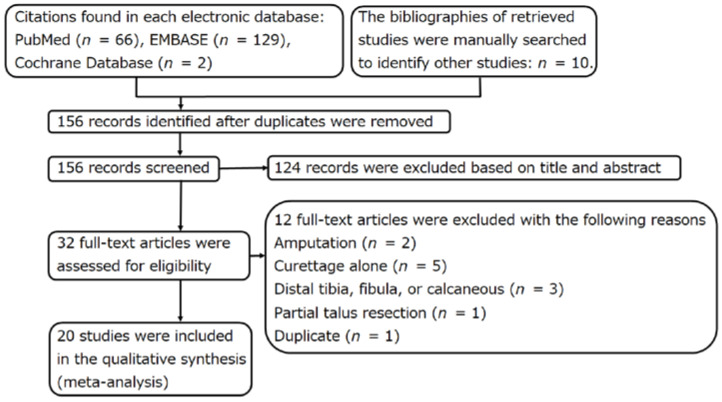
Flow chart of the search for relevant articles.

Of the 156 studies identified in the search, 20 were finally included (Figure 1; Table 1, Table 2 and Table 3) [7,8,9,10,11,12,13,17,18,19,20,21,22,23,24,25,27,28,29,30]. There were no RCTs among them.

### 2.7. Demographic Data and Ratio of Patients Who Underwent Reconstruction with Tibiocalcaneal Fusion, Frozen Autograft, or Talar Prosthesis

A total of 23 patients who underwent reconstruction after the resection of tumors originating in the talus were included. Of them, 17 (74%) underwent reconstruction with tibiocalcaneal fusion, 1 (4%) with a frozen autograft, and 5 (22%) with a talar prosthesis (Table 1, Table 2 and Table 3).

### 2.8. Methodological Quality of Included Studies

Evaluation of the quality of the individual studies using the RoBANS tool showed an overall moderate risk of bias. All 20 included studies showed that “selection of participants” and “confounding variables” were high, whereas “measurement of exposure,” “blinding of outcome,” “incomplete outcome data,” and “selective outcome reporting” were low.

## 3. Results

The mean ankle joint ROM in plantar/dorsiflexion in the tibiocalcaneal fusion group was 32.5 (range 30–35) [18,19], while that of the talar prosthesis group was 36.7 (range 25–45; Table 3) [11,24,30]. The mean AOFAS score in the tibiocalcaneal fusion group was 77 (range 71–83) [25,28], while that of the talar prosthesis group was 89 (range 86–91) [11,24,30]. The AOFAS score was higher in the group reconstructed using a talar prosthesis (Table 3). The mean MSTS score in the tibiocalcaneal fusion group was 77.6 (range 66–90) [7,9,13,25], that in the frozen autograft group was 70 [8], and that in the talar prosthesis group was 90 (range 87–93) [12,30]. The MSTS score was higher in the talar prosthesis group than in the tibiocalcaneal fusion or frozen autograft groups (Table 3).

The mean leg length difference was 0.4 cm (range 0–1.5 cm) in the tibiocalcaneal fusion group [10,13,18,28] and 0 cm in the talar prosthesis and frozen autograft groups (Table 3) [8,11,12,23,24,30]. The mean leg length difference in the tibiocalcaneal fusion group treated with an autograft or allograft was 0.2 cm (range 0–1 cm) [10,13,28], whereas that in the tibiocalcaneal fusion group treated without an autograft or allograft was 1.5 cm [18] (Table 2).

Regarding complications, in the group of patients who underwent tibiocalcaneal fusion, a partial sensory deficit in the distribution of the medial dorsal cutaneous nerve was observed in 1 patient (6%) versus venous thrombosis in 2 (12%) [7,9,10,13,17,18,19,20,21,22,25,27,28,29] (Table 3). In the group reconstructed with frozen autografts, osteoarthritic changes were observed in 1 patient (100%) [8] (Table 3). No complications occurred in the talar prosthesis group [11,12,23,24,30] (Table 3).

The mean age of the tibiocalcaneal fusion, frozen autograft, and talar prosthesis groups was 30.3 (range 15–62) [7,9,10,13,17,18,19,20,21,22,25,27,28,29], 15 [8], and 31 (range 22–43) years [11,12,23,24,30], respectively. The mean patient age was lower in the frozen autograft group than in the other groups (Table 3). The proportion of men in the tibiocalcaneal fusion, frozen autograft, and talar prosthesis groups was 65% [7,9,10,13,17,18,19,20,21,22,25,27,28,29], 100% [8], and 60% [11,12,23,24,30], respectively. There was a higher percentage of men in the frozen autograft group than in the other groups (Table 3). The proportion of malignant tumors in the tibiocalcaneal fusion, frozen autograft, and talar prosthesis groups was 35% [7,9,10,13,17,18,19,20,21,22,25,27,28,29], 100% [8], and 60% [11,12,23,30,34], respectively. The proportion of malignant tumors was higher in the frozen autograft and talar prosthesis groups (Table 3). A total of 3 patients (18%) in the tibiocalcaneal fusion group underwent resection of the peritalar bone along with the talar tumor [7,10,25] versus no patients in the talar prosthesis or frozen autograft group (0%) (Table 3). The proportion of continuous disease free as the oncologic outcomes of the tibiocalcaneal fusion, frozen autograft, and talar prosthesis groups was 76% [7,9,10,13,17,18,19,20,21,22,25,27,28,29], 100% [8], and 80% [11,12,23,24,30], respectively. The oncological outcomes were similar among groups (Table 3). The mean postoperative follow-up period in the tibiocalcaneal fusion, frozen autograft, and talar prosthesis groups was 4.2 (range 0.5–17) [7,9,10,13,17,18,19,20,21,22,25,27,28,29], 5 [8], and 1.6 (range 0.5–3.5) years [11,12,23,24,30], respectively. The postoperative follow-up period was shorter for the talar prosthesis group than the tibiocalcaneal fusion and frozen autograft groups (Table 3).

## 4. Discussion

Until 2008, tibiocalcaneal fusion was the only reported reconstruction method after talar tumor resection [7,17,18,19,20,25,27]. In 2009, Sakayama et al. reported a reconstruction method using frozen autografts [8]. In 2014, Harnroongroj et al. reported a reconstruction method using a talar prosthesis [23]. We analyzed studies that reported on the postoperative function and complications after reconstruction following talar tumor resection and compared the postoperative function and risk of complications among the tibiocalcaneal fusion, frozen autograft, and talar prosthesis groups. The talar prosthesis group had a shorter follow-up period but better postoperative function than the other groups. The risk of complications was higher with tibiocalcaneal fusion and frozen autograft reconstruction than with talar prosthesis reconstruction. Therefore, talar prosthesis reconstruction seems preferable to conventional methods, such as tibiocalcaneal fusion, after talar tumor resection.

This study had several limitations. First, all included studies were retrospective and had a patient-background bias. Because talar prosthesis is a relatively new reconstruction method, its postoperative follow-up period was shorter than those of the other reconstruction methods. Although the talar prosthesis reconstruction method showed better postoperative function than the other reconstruction methods, it will be necessary to analyze long-term follow-up data in the future. Harnroongroj et al. used a talar body prosthesis to replace the talus in 33 patients with talar osteonecrosis, talar fracture, or talar tumor [23]. They reported that the AOFAS score of the 10–20 years (*n* = 8), 20–30 years (*n* = 11), and 30–36 years (*n* = 9) follow-up groups was 78, 76, and 76, respectively, which indicated that talar body prosthesis can maintain good function over a long follow-up period [23]. Second, a higher percentage of patients in the tibiocalcaneal fusion group underwent resection of the peritalar bone and the talar tumor. This may have affected the functional outcomes. Reconstruction with a talar prosthesis cannot be performed in patients for whom the peritalar bone must be resected. Therefore, its use is limited to reconstruction after the resection of malignant tumors localized within the talus or intermediate-grade tumors. In contrast, tibiocalcaneal fusion can be performed even in cases in which the peritalar bone must be resected, and its use is more widely indicated than talar prosthesis reconstruction. RCTs can avoid many of these biases by randomly allocating participants to groups. Since no RCTs were identified, well-designed cohorts and observational studies with strong effects may provide reliable information. Third, patients who underwent resection and reconstruction for talar tumors were rare, resulting in a small number of patients being analyzed (only 23 patients). However, at present, this is all the information we have on reconstruction after talar tumor resection, and the results of this systematic review will be useful to orthopedic surgeons treating tumors.

These results suggest that patients who underwent talar prosthesis reconstruction had better postoperative function than those who underwent other reconstruction methods. Tibiocalcaneal fusion is a widely accepted reconstruction method after talar tumor resection; however, it sacrifices the ankle joint ROM and results in a leg length discrepancy due to shortening unless combined with bone grafting [7,9,10,13,17,18,19,20,21,22,25,27,28,29]. Bussewitz et al. performed tibiocalcaneal fusion using an allogeneic femoral head bone graft and intramedullary nail in 25 patients with a mean follow-up period of 83 (range 10–265) weeks and achieved bone fusion in 12 (48%) [34]. The mean time to full weight bearing was 117.7 (range 43–209) days [34]. Tenenbaum et al. performed tibiotalocalcaneal fusion with an intramedullary nail without bone grafting in 14 patients with talus osteonecrosis, followed them for a mean 26.1 (range 12–47) months, and reported a mean AOFAS score of 72.1 (range 46–86) [35].

The talar prosthesis maintains ankle joint motion and leg length [23,36]. This is because the implant is usually modeled from a computed tomography scan of the contralateral talus and is anatomically correct for mortise [36]. In addition, reconstruction with a talar prosthesis has a shorter time to full weight bearing (5 weeks) [36]. Taniguchi et al. [36] replaced 55 talus osteonecroses of 51 patients with a total talar prosthesis. They reported that the Japanese Society for Surgery of the Foot ankle-hindfoot scale [37,38] score was a mean 89.4 (range 76–100) after a mean follow-up of 52.8 months [36]. Thereafter, Morita et al. reported that among 18 patients (19 talus) with more than 10 years of follow-up (median 152 months), the median Japanese Society for Surgery of the Foot ankle-hindfoot scale was 97 (interquartile range 87–99.5) [39].

This study revealed that the risk of complications was higher after tibiocalcaneal fusion and frozen autograft than after talar prosthesis reconstruction. Tenenbaum et al. performed tibiotalocalcaneal fusion with intramedullary nails without bone grafting in 14 patients with talus osteonecrosis and noted complications of fatigue fracture in 1 patient (7%), hardware removal in 3 (21%), and superficial infection in 1 (7%) [35]. Hayashi et al. performed osteoarticular frozen autograft reconstruction in 27 patients with bone tumors, including epiphyses, and reported that grade IV osteoarthritis occurred in 12 patients (44%) and infection in 6 (22%) after a mean follow-up of 94.0 months [40].

Harnroongroj et al. reported that talar body prosthesis replacement of the talus in 33 patients with talar osteonecrosis, talar fracture, and talar tumor resulted in a size discrepancy in 2 patients (6%), infection in 1 (3%), and osteonecrosis of the talar head and neck in 1 (3%) [23]. Taniguchi et al. reported that 22 patients with osteonecrosis of the talus underwent replacement with a talar body prosthesis and were followed up for a mean of 98 (range 18–174) months. Of the 22 patients, 4 (18%) sustained fractures of the talar head and neck that required revision with a total talar prosthesis [41]. Taniguchi et al. [36] replaced 55 taluses of 51 patients with talus osteonecrosis with a total talar prosthesis. After a mean follow-up of 52.8 months, a radiographic evaluation revealed osteosclerosis of the distal tibia in 44%, the navicular bone in 9%, and the calcaneus in 35%; however, no complications required revision surgery [36]. Thereafter, Morita et al. reported that in 18 patients (19 talus) with more than 10 years of follow-up (median 152 months), degenerative changes were observed in the distal tibia in 90%, the navicular bone in 16%, and in the calcaneus in 11%; however, no complications required revision surgery [39]. Thus, they recommend use of a total talar prosthesis rather than a talar body prosthesis [41].

We propose a treatment strategy for talar tumors using Enneking stage (Table 4) [42]. For stage 3 tumors, intermediate-grade tumors with large extraskeletal lesions, pathologic fractures with intra-articular involvement, or complex fractures, we recommend marginal resection followed by total talar prosthesis reconstruction (Table 4). Because the articular cartilage acts as a barrier to tumor invasion, malignant tumors rarely penetrate the articular cartilage into the joint and often spread extraskeletally along the ligamentous and articular capsule attachments [43,44,45,46].

**Figure 2:**
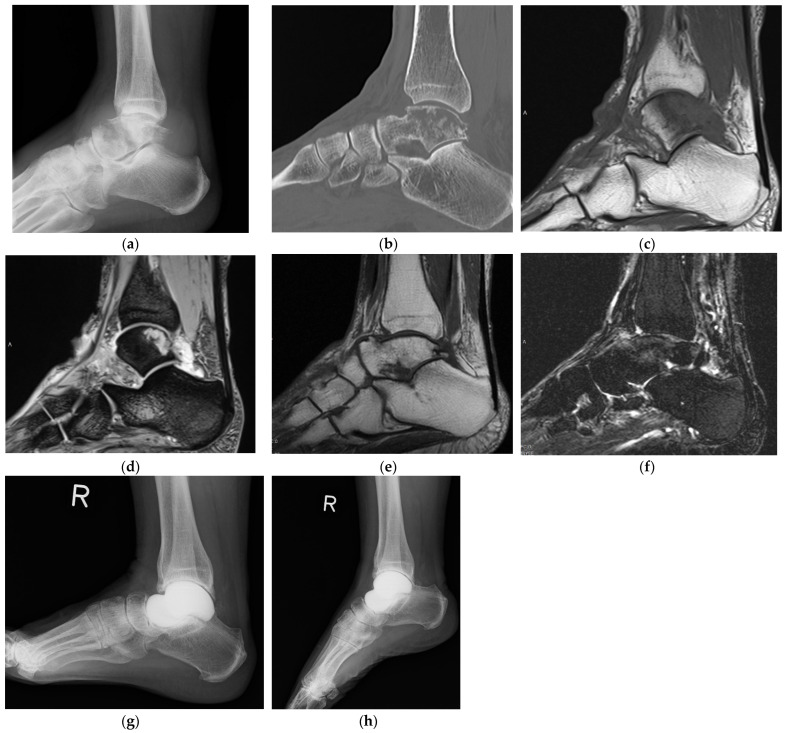
Primary diffuse large B-cell lymphoma of the talus (stage IA) in a 74-year-old woman. The patient presented with a chief complaint of ankle pain and swelling. (**a**) Radiography showed osteolytic lesions and talar body collapse. Soft tissue shadows were also seen anterior and posterior to the ankle joint. (**b**) Computed tomography showed diffuse osteolytic lesions and talar body collapse. (**c**,**d**) Magnetic resonance imaging (MRI) showed low-signal-intensity lesions on T1WI and high-signal-intensity lesions on T2WI in the posterior part of the talar body and the extraskeletal area (**c**: T1WI; **d**: T2WI). A needle biopsy was performed, and a diagnosis of diffuse large B-cell lymphoma was made. (**e**,**f**) After six courses of rituximab, cyclophosphamide, doxorubicin, vincristine, and prednisolone, MRI showed a marked reduction in tumor size (**e**: T1WI; **f**: T2WI); however, the patient had severe ankle joint pain due to the talar body collapse that was treated with talar tumor resection and total talar prosthesis reconstruction [36,47]. (**g**,**h**) Six years after surgery, osteosclerotic changes were observed in the distal tibia and calcaneus; however, the patient was able to walk unaided without pain. No tumor recurrence was observed (**g**: dorsiflexed position; **h**: plantar flexed position).

Quan et al. reported that all 11 patients with osteosarcoma but without evidence of intra-articular invasion on preoperative magnetic resonance imaging (MRI) were free of intra-articular invasion upon histological evaluation [43]. If there is no evidence of intra-articular invasion on a preoperative MRI, the tumor can be removed by intra-articular resection [43], followed by total talar prosthesis reconstruction (Stage IA and IIA; Table 4; Figure 2). Malignant talar tumors with intra-articular invasion or extraskeletal involvement that require resection of the peritalar bone cannot be reconstructed with total talar prosthesis and usually require tibiotalocalcaneal fusion (Stage IB and IIB; Table 4). Bone grafting should be used to correct leg length differences. If the talar tumors cannot be removed with negative margins, below-the-knee amputation should be indicated (Stage IB and IIB; Table 4) [1].

## 5. Conclusions

In conclusion, after talar tumor resection, talar prosthesis reconstruction features better postoperative function than tibiocalcaneal fusion or frozen autograft reconstruction. The risk of complications was higher for tibiocalcaneal fusion or frozen autograft reconstruction than for talar prosthesis reconstruction. Therefore, talar prosthesis reconstruction appears preferable to traditional methods, such as tibiocalcaneal fusion reconstruction after talar tumor resection. However, because this systematic review included only retrospective studies with a small number of patients, its results require re-evaluation in future RCTs with larger numbers of patients.

## Figures and Tables

**Table 1:** Characteristics of included studies.

Author and Year	Study Type	Age (y)	Sex	Histology	Reconstruction Method	Details
Mechlin 1984 [17]	Case report	18	F	GCTB	Tibiocalcaneal fusion	Talectomy and tibiocalcaneal fusion with iliac bone graft
Sanjay 1992 [18]	Case report	34	F	GCTB	Tibiocalcaneal fusion	Talectomy and tibiocalcaneal fusion
Itokazu 1994 [19]	Case report	30	F	Osteoblastoma	Tibiocalcaneal fusion	Talectomy and tibiocalcaneal fusion
Ogose 1995 [27]	Case report	33	F	Clear cell chondrosarcoma	Tibiocalcaneal fusion	Talectomy and tibiocalcaneal fusion
Luna 2007 [25]	Case report	27	M	Epithelioid hemangioma	Tibiocalcaneal fusion	Excision of the talus, navicular, and portions of the medial cuneiform, with midfoot and hindfoot fusion using an allograft
Ramdas 2007 [20]	Case report	15	M	GCTB	Tibiocalcaneal fusion	Talectomy and tibiocalcaneal fusion
Katagiri 2008 [7]	Case report	49	M	Osteosarcoma	Tibiocalcaneal fusion	Excision of the talus, navicular, portion of distal tibia and calcaneous. Tibiocalcaneal fusion with iliac bone graft. Free latissimus dorsi flap.
Sharma 2009 [21]	Case report	19	M	GCTB	Tibiocalcaneal fusion	Talectomy and tibiocalcaneal fusion
Sakayama 2009 [8]	Case report	15	F	Osteosarcoma	Frozen autograft	Talectomy and an osteoarticular frozen autograft
Wang 2011 [9]	Case report	21	F	Osteosarcoma	Tibiocalcaneal fusion	Talectomy and tibiocalcaneal fusion with allograft
Kumar 2014 [22]	Case report	62	M	GCTB	Tibiocalcaneal fusion	Talectomy and tibiocalcaneal fusion
Harnroongroj 2014 [23]	Case report	27	M	GCTB	Talar prosthesis	Talectomy and talar body prosthesis (stainless steel, constrained)
Bahamonde 2017 [28]	Case report	27	M	Chondroblastoma	Tibiocalcaneal fusion	Talectomy and tibiocalcaneal fusion with fibula autograft
Vosoughi 2017 [29]	Case report	25	M	ABC	Tibiocalcaneal fusion	Talectomy and tibiocalcaneal fusion with fibula autograft
Kamal 2018 [10]	Case report	38	M	Osteosarcoma	Tibiocalcaneal fusion	Excision of the talus, distal tibia, and distal fibula; triple arthrodesis using fibula autograft and allograft
Fang 2018 [30]	Case report	43	F	Mesenchymal sarcoma	Talar prosthesis	Talectomy and total talar prosthesis (titanium and UHMWPE, constrained)
Papagelopoulos 2019 [11]	Case report	30	F	Ewing sarcoma	Talar prosthesis	Talectomy and total talar prosthesis combined with tibia component (titanium nitride, constrained)
Huang 2021 [12]	Case report	31	M	Osteosarcoma	Talar prosthesis	Talectomy and total talar prosthesis (titanium, nonconstrained)
Rosli 2021 [13]	Case report	30	M	ABC	Tibiocalcaneal fusion	Talectomy and tibiocalcaneal fusion with triangular double-barrel free fibula flap
Rosli 2021 [13]	Case report	39	F	Osteosarcoma	Tibiocalcaneal fusion	Talectomy and tibiocalcaneal fusion with triangular double-barrel free fibula flap
Rosli 2021 [13]	Case report	34	M	Osteosarcoma	Tibiocalcaneal fusion	Talectomy and tibiocalcaneal fusion with triangular double-barrel free fibula flap
Rosli 2021 [13]	Case report	27	M	Chodromyxoid fibroma	Tibiocalcaneal fusion	Talectomy and tibiocalcaneal fusion with triangular double-barrel free fibula flap
Yang 2021 [24]	Case report	22	M	GCTB	Talar prosthesis	Talectomy and total talar prosthesis (ocrmo material, constrained)

ABC, aneurysmal bone cyst; F, female; GCTB, giant cell tumor of the bone; M, male; UHMWPE, ultra-high molecular weight polyethylene; y, years.

**Table 2:** Additional characteristics of included studies.

Author and Year	ROM of Plantar/ Dorsiflexion	AOFAS	MSTS	Limb Length Discrepancy (cm)	Complications	Oncological Outcomes	Follow-Up Period (Year)
Mechlin 1984 [17]	NR	NR	NR	NR	None	CDF	0.75
Sanjay 1992 [18]	35	NR	NR	1.5	None	CDF	3.5
Itokazu 1994 [19]	30	NR	NR	NR	None	CDF	17
Ogose 1995 [27]	NR	NR	NR	NR	None	Local recurrence, BK, NED	13
Luna 2007 [25]	NR	71	76	NR	Sensory deficit *	CDF	5
Ramdas 2007 [20]	NR	NR	NR	NR	None	CDF	0.5
Katagiri 2008 [7]	NR	NR	90	NR	None	CDF	3.9
Sharma 2009 [21]	NR	NR	NR	NR	None	CDF	1.5
Sakayama 2009 [8]	NR	NR	70	0	Osteoarthritis	CDF	5
Wang 2011 [9]	NR	NR	86	NR	None	CDF	1.6
Kumar 2014 [22]	NR	NR	NR	NR	None	Local recurrence, BK, lung metastases: stable	2
Harnroongroj 2014 [23]	NR	NR	NR	0	None	Local recurrence (malignant change), BK, NED	1
Bahamonde 2017 [28]	NR	83	NR	1	None	CDF	11
Vosoughi 2017 [29]	NR	NR	NR	NR	None	CDF	1.5
Kamal 2018 [10]	NR	NR	NR	0	None	CDF	1
Fang 2018 [30]	40	91	87	0	None	CDF	0.5
Papagelopoulos 2019 [11]	45	86	NR	0	None	CDF	3.5
Huang 2021 [12]	NR	NR	93	0	None	CDF	2
Rosli 2021 [13]	NR	NR	76	0	Venous thrombosis	CDF	11
Rosli 2021 [13]	NR	NR	73	0	None	AWD (lung metastases)	6
Rosli 2021 [13]	NR	NR	76	0	None	DOD	4
Rosli 2021 [13]	NR	NR	66	0	Venous thrombosis	CDF	2
Yang 2021 [24]	25	89	NR	0	None	CDF	1

* A partial sensory deficit in the distribution of the medial dorsal cutaneous nerve. AOFAS, American Orthopedic Foot and Ankle Society; AWD, alive with disease; BK, below-the-knee amputation; CDF, continuous disease free; DOD, death of disease; MSTS, Musculoskeletal Tumor Society; NED, no evidence of disease; NR, not reported; ROM, range of motion; y, years.

**Table 3:** Summary of functional outcomes and complications for each reconstruction method.

Reconstruction Type	Mean Age (Range)	Sex	Histology	Resection of Bone Around Talus, *n* (%)	Mean (Range) ROM of Plantar/Dorsiflexion	Mean (Range) AOFAS Score	Mean (Range) MSTS score	Mean Limb Length Discrepancy (Range)	Complication	Oncological Outcome	Mean (Range) Follow-Up Period
Tibiocalcaneal fusion (*n* = 17)	30.3 (15–62)	M: 11 (65%), F: 6 (35%)	Benign: 4 (24%); Intermediate: 7 (41%); Malignant: 6 (35%)	3 (18%)	32.5 (30–35)	77 (71–83)	77.6 (66–90)	0.4 cm (0–1.5)	Sensory deficit: 1 (6%), Venous thrombosis: 2 (12%)	CDF: 13 (76%), NED: 1 (6%), AWD: 2 (12%), DOD: 1 (6%)	4.2 years (0.5–17)
Frozen autograft (*n* = 1)	15	F: 1 (100%)	Malignant: 1 (100%)	0	NR	NR	70	0 cm	None (0%)	CDF: 1 (100%)	5 years
Talar prosthesis (*n* = 5)	31 (22–43)	M: 3 (60%), F: 2 (40%)	Intermediate: 2 (40%), Malignant: 3 (60%)	0	36.7 (25–45)	89 (86–91)	90 (87–93)	0 cm	None (0%)	CDF: 4 (80%), NED: 1 (20%),	1.6 years (0.5–3.5)

AOFAS, American Orthopaedic Foot and Ankle Society; AWD, alive with disease; CDF, continuous disease free; DOD, death of disease; F, female; M, male; MSTS, Musculoskeletal Tumor Society; NED, no evidence of disease; NR, not reported; ROM, range of motion.

**Table 4:** Treatment strategy for talar tumors.

Enneking Stage [42]	Treatment
1	Observation
2	Curettage combined with local adjuvant therapy and cement or bone graft
3	Curettage combined with local adjuvant therapy and cement or bone graft Marginal resection and total talar replacement
IA	Intra-articular resection and total talar replacement
IB	Wide resection and arthrodesis Below-the-knee amputation
IIA	Intra-articular resection and total talar replacement
IIB	Wide resection and arthrodesis Below-the-knee amputation

## Supplementary Materials

The following are available online at https://www.mdpi.com/article/10.3390/curroncol29120769/s1: Table S1: Search strategy.
