# Supplementary material for: Reconstruction after Talar Tumor Resection: A Systematic Review
Source: Curr Oncol. 2022 Dec 12;29(12):9788–800. doi: 10.3390/curroncol29120769 (PMC9777178; doi:10.3390/curroncol29120769)
Supplement: Supplementary file 1 [file curroncol-29-00769-s001.zip › curroncol-2068869-supplementary.pdf]

Supplementary material Table S1: Search strategy.

2022/1/19

| # PubMed | Literature search | Result |
|---|---|---|
| 1 | talus[tw] OR talar[tw] | 8,530 |
| 2 | tumo*[tw] OR cancer*[tw] OR neoplasm*[tw] OR osteosarcoma*[tw] OR osteoblastoma*[tw] OR osteochondroma*[tw] OR chondroblastoma*[tw] OR chondrosarcoma*[tw] OR chondroma*[tw] OR oncologic*[tw] | 4,331,033 |
| 3 | #1 and #2 | 501 |
| 4 | "Bone Neoplasms/surgery"[mh] AND "Talus/surgery"[mh] | 66 |
| 5 | #3 or #4 | 501 |
| 6 | "Reconstructive Surgical Procedures"[mh] OR reconstruct*[tw] OR repair*[tw] OR prosthe*[tw] OR replace*[tw] OR substitute*[tw] | 1,621,155 |
| 7 | #5 and #6 | 66 |

Embase 2022/1/19

| Set# | Searched for | Results |
|---|---|---|
| S1 | (talus OR talar) | 11958* |
| S2 | (tumo* OR cancer* OR neoplasm* OR osteosarcoma* OR osteoblastoma* OR osteochondroma* OR chondroblastoma* OR chondrosarcoma* OR chondroma* OR oncologic*) | 6844161* |
| S3 | (S1 and S2) | 994° |
| S4 | EMB.EXACT.EXPLODE("talus") AND (EMB.EXACT.EXPLODE("bone tumor -- surgery")) | 168° |
| S5 | (S3 or S4) | 1006° |
| S6 | (EMB.EXACT.EXPLODE("reconstructive surgery")) OR (reconstruct* OR repair* OR prosthe* OR replace* OR substitute*) | 2215667* |
| S7 | (S5 and S6) | 129° |

Central 2022/1/19

| ID | Search | Hits |
|---|---|---|
| #1 | (talus OR talar):ti,ab,kw | 259 |
| #2 | (tumo* OR cancer* OR neoplasm* OR osteosarcoma* OR osteoblastoma* OR osteochondroma* OR chondroblastoma* OR chondrosarcoma* OR chondroma* OR oncologic*):ti,ab,kw | 222703 |

| | | |
|---|---|---|
| #3 | #1 and #2 | 8 |
| #4 | [mh "Bone Neoplasms"/SU] AND [mh "Talus"/SU] | 0 |
| #5 | #3 or #4 | 8 |
| #6 | ([mh "Reconstructive Surgical Procedures"]) OR ((reconstruct* OR repair* OR prosthe* OR replace* OR substitute*):ti,ab,kw) | 82247 |
| #7 | #5 and #6 | 2 |
| #8 | #5 and #6 in Cochrane Reviews, Cochrane Protocols | 0 |
| #9 | #5 and #6 in Trials | 2 |
